# Atrial Tachycardia Utilizing Bachmann's Bundle as an Epicardial Bypass: A Case Suggestive of a Dual‐Loop Mechanism

**DOI:** 10.1111/anec.70240

**Published:** 2026-09-29

**Authors:** Javeria Anees

**Affiliations:** ^1^ Bolan Medical College Quetta Pakistan

Dear Editor,

I read with considerable interest the recent case report by Sakurai et al., published in the *Annals of Noninvasive Electrocardiology*, describing a novel presentation of post‐ablation atrial tachycardia (AT) in which Bachmann's bundle was proposed to function as an epicardial bypass within both roof‐dependent and localized‐reentrant circuits—a mechanistic constellation reported as previously undescribed in the literature (Sakurai et al. 2026). This case reflects a broader unresolved challenge: Christian‐Miller et al. recently demonstrated that Bachmann's bundle‐related atrial tachycardias remain poorly characterized at a field level precisely because their epicardial location renders them inaccessible to standard endocardial mapping protocols (Christian‐Miller et al. 2026).

While we commend the scientific novelty of this report, we wish to draw attention to a fundamental methodological limitation that materially undermines the validity of its central conclusion. The identification of Bachmann's bundle as the responsible epicardial bypass rests exclusively on indirect endocardial evidence: widespread centrifugal breakthrough activation across the anterior wall and fragmented electrograms at the breakthrough site. No direct epicardial mapping was undertaken, and orthodromic capture of the breakthrough site during entrainment pacing could not be established, as the authors themselves acknowledge. Critically, neither finding is specific to Bachmann's bundle. Centrifugal breakthrough patterns may arise equally from the septopulmonary bundle or residual myocardial fiber tracts overlying fibrotic substrate, while fragmented potentials are well‐recognized signatures of heterogeneous conduction within scarred myocardium, independent of any specific epicardial structure (Nakatani et al. 2021; Nayak et al. 2020). Furthermore, the dual‐loop mechanistic characterization—the manuscript's central novel claim—rests on circuit geometry inferred entirely from endocardial activation sequences, without entrainment confirmation of both loop components independently. The structural identity of the bypass was therefore inferred on the basis of anatomical probability alone, rather than established by electrophysiological confirmation or imaging correlation. This distinction carries direct clinical consequence: the precise structure mediating epicardial bypass conduction directly governs ablation strategy, target site selection, and anticipated recurrence risk. Notably, the very source cited in support of this inference explicitly cautions that such characterization requires direct epicardial access—an inconsistency the manuscript does not resolve (Nakatani et al. 2021). We propose that future cases implicating a specific epicardial bypass structure should satisfy at least two of the following: direct epicardial mapping with activation confirmation, entrainment with orthodromic capture at the breakthrough site, pre‐procedural CT angiographic localization of Bachmann's bundle, or demonstrated sequential LA/RA ablation dependence. Such a framework would provide reproducible evidentiary standards to guide both mechanistic interpretation and ablation strategy in this challenging substrate (Suzuki et al. 2025; Christian‐Miller et al. 2026).

The rigorous distinction between inferential and confirmatory evidence remains indispensable if case‐report‐derived conclusions are to responsibly guide electrophysiology practice.

## Author Contributions

The author contributed to the conception, literature review, writing, revision, and final approval of this manuscript.

## Funding

The author has nothing to report.

## Ethics Statement

The author has nothing to report.

## Conflicts of Interest

The author declares no conflicts of interest.

## Data Availability

The author has nothing to report.
